# Molecular Mechanism and Energy Basis of Conformational Diversity of Antibody SPE7 Revealed by Molecular Dynamics Simulation and Principal Component Analysis

**DOI:** 10.1038/srep36900

**Published:** 2016-11-10

**Authors:** Jianzhong Chen, Jinan Wang, Weiliang Zhu

**Affiliations:** 1 School of Science, Shandong Jiaotong University, Jinan, 250014, China; 2 Drug Discovery and Design Center, CAS Key Laboratory of Receptor Research, Shanghai Institute of Materia Medica, Chinese Academy of Sciences, 555 Zuchongzhi Road, Shanghai, 201203, China

## Abstract

More and more researchers are interested in and focused on how a limited repertoire of antibodies can bind and correspondingly protect against an almost limitless diversity of invading antigens. In this work, a series of 200-ns molecular dynamics (MD) simulations followed by principal component (PC) analysis and free energy calculations were performed to probe potential mechanism of conformational diversity of antibody SPE7. The results show that the motion direction of loops H3 and L3 is different relative to each other, implying that a big structural difference exists between these two loops. The calculated energy landscapes suggest that the changes in the backbone angles ψ and φ of H-Y101 and H-Y105 provide significant contributions to the conformational diversity of SPE7. The dihedral angle analyses based on MD trajectories show that the side-chain conformational changes of several key residues H-W33, H-Y105, L-Y34 and L-W93 around binding site of SPE7 play a key role in the conformational diversity of SPE7, which gives a reasonable explanation for potential mechanism of cross-reactivity of single antibody toward multiple antigens.

Invasions of antigens into human body may generate serious damage toward organism of human. In response, human body can trigger immunological reaction and produce antibodies to turn against pathogenic antigens^1^,^2^. Ongoing researches have shown that the number of antibodies in the primary response is finite, while antigen space is infinite^3^,^4^. This fact raises a fundamental question: how can a limited repertoire of antibodies bind and correspondingly protect against an almost limitless diversity of invading antigens. To reasonably explain this issue, Pauling proposed that specific binding sites should be sought out of an ensemble of preexisting antibody conformations^5^. This rational proposal indicates that each antibody can bind to more than one antigen or cross-react with multiple antigens^6^,^7^,^8^,^9^,^10^,^11^. Thus, it is essential to probe the details involving molecular mechanism of antibody conformational diversity for understanding the central role that cross-reactivity of antibodies plays in autoimmunity and allergy^12^,^13^,^14^.

To date, crystal structures of multiple antibodies complexed with antigens and haptens have been determined^15^,^16^,^17^,^18^, which provides structural basis for further insight into the relationship of single antibody toward multiple antigens or cross-reactivity of antibodies. These existed structures suggest that the cross-reactivity of antibodies can be achieved by the shared ligand chemistry or molecular mimicry^19^,^20^,^21^. For example, an antibody toward HIV-1 protein P24 can also bind with other unrelated peptides using the same binding sites as the protein P24^22^. The antibody D1.3 toward lysozyme not only strongly binds to lysozyme, but also efficiently protects against an anti-idiotype antibody^23^. These studies show that antibodies can adjust their conformations by rearranging the side chains of several residues to accept different ligands, which means that multiple antigens or haptens can fit into a single antibody-binding site^24^,^25^,^26^,^27^,^28^.

The previous studies demonstrated that the conformations of many antibodies in *apo* and bound states is obviously different^28^,^29^,^30^,^31^. For example, the antibody SPE7 studied by Tawfik *et al*., a monoclonal immunoglobulin E (IgE) against a 2,4-dinitrophenyl (DNP) hapten, displays four different conformations in its *apo* and bound situations. In the *apo* state, the heterodimer of SPE7 exhibits two different conformations (termed Ab1 and Ab2, respectively). In the alizarin red (AZR)-SPE7 complex, the binding of AZR induces the third antibody conformation (called Ab3), while the association of SPE7 with a recombinant protein antigen (Trx-Shear3) leads to the fourth conformation (termed Ab4). Four different conformations of SPE7 are shown in Fig. 1 in surface modes and structures of AZR and Trx-Shear3 are displayed in support information (Figure S1A and B). As shown in Fig. 1, the Ab1 conformation exhibits a flatter and more regular channel (Fig. 1A), but the Ab2 conformation is funnel-shaped and terminated in a deep pocket (Fig. 1B). Figure 1C shows that the Ab3 conformation displays a foot-shaped and deep pocket. The Ab4 conformation is similar to the Ab1, but the Ab4 has a relatively flat binding site with a truncated channel. These different conformations are mainly shaped by the residues H-W33, H-Y101 and H-Y105 in the chain H and L-Y34 and L-W93 in the chain L. These residues build two important loops H3 (the third loop in the chain H) and L3 (the third loop in the chain L), which are displayed in Figure S1C. The work from Tawfik *et al*. revealed a large conformational difference in the loops H3 and L3 induced by large sidechain alterations of several key residues, which may imply an underlining important and interesting mechanism of the relationship of single antibody toward multiple antigens. Thus, it is significant to reveal molecular mechanism and energy basis of SPE7 conformational diversity at an atomic level for understanding cross-reactivity of antibodies and antibody–drug conjugated anti-disease treatment.

Molecular dynamics (MD) simulations play an important role in successful exploration of protein-ligand interactions and conformational changes of proteins at the atomic level^32^,^33^,^34^,^35^,^36^,^37^,^38^,^39^,^40^,^41^,^42^,^43^. Principal component (PC) analysis^44^,^45^,^46^,^47^, an important method of post-process analysis based on trajectories of MD simulations, shows a big potential in probing internal dynamics of protein motions^48^,^49^. In this work, a combination of MD simulations and PC analysis is applied to investigate the molecular mechanism of antibody conformational diversity and correlated motion of antibody. Free energy landscapes based on trajectories of MD simulations also provide detailed information related with conformational changes of proteins^50^,^51^,^52^. Thus, free energy landscapes between multiple physical quantities from MD trajectory are calculated to reveal related energetic information of antibody conformational diversity. We expect that this study can contribute a significant theoretical hint to antibody-drug conjugated anti-disease treatment.

## Results

### Dynamics analysis of conformational diversity

To evaluate structural deviation of four conformations relative to their crystal structures, distributions of RMSD of backbone atoms in SPE7 were calculated (Figure S2). RMSDs of two conformations Ab1 and Ab2 are mostly distributed at 1.30 and 2.49 Å, respectively. RMSD of the conformation Ab3 mainly focuses on 1.10 and 1.50 Å, while the one of the conformation Ab4 is located at 1.50 and 1.71 Å. These results suggest that four conformations produce different changes relative to their crystal structures. Free energy landscapes built using RMSD and gyration radius were depicted in Figure S3. The results prove that four conformations of SPE7 embody different energy levels.

Root-mean-square fluctuations (RMSF) of C_α_ atoms can provide direct insight into the structural fluctuation and flexibility of proteins. RMSF values of C_α_ atoms in four different conformations of SPE7 were computed and shown in Fig. 2. Except for the two terminus, one can observe that RMSFs of the regions near residues 30, 42, 56, 104, 147, 161 and 178 of SPE7 produce obvious changes. These changes in RMSF reflect that the flexibilities of four different conformations are different from each other, which basically embodies a cause of SPE7 conformational diversity.

To further probe internal dynamics of SPE7 conformational diversity, general cross-correlation analysis was carried out using the method developed by Lange *et al*.^53^ and the results were depicted in Fig. 3. Four conformations exhibit obvious difference in correlated extents of protein motion. Totally, the Ab1 and Ab3 conformations display strong correlated motions (red and yellow in Fig. 3A,C), while the Ab2 and Ab4 conformations are embodied by low correlated motion (blue in Fig. 3B,D). In the case of the Ab1, the loop H3 (R3 region) and the R4 region from the residues 149 to 175 (near L-Y34) generate strong correlated motion (Fig. 3A). Additionally, the loop H3 moves in a strong correlated mode relative to the residues 1–40 of the chain H, and the loop L3 (R4 region) also produces a strong correlated motion relative to the residues 122–162 of the chain L. Compared to the Ab1, the Ab3 slightly weakens the correlated motion. However, the correlated motions in the Ab2 and Ab4 are heavily reduced, especially for the Ab4. The above regions relating with obvious changes of motion modes agree with the previous RMSF changes of SPE7. These changes of internal dynamics may reflect different alternation of relative positions between key residues, which may play an important role in conformational diversity of SPE7.

### Principal component analysis

To understand motion changes of four conformations in more details, PC analysis, a powerful tool to probe conformational changes of proteins^54^,^55^, was performed on MD trajectories. The eigenvalues were obtained by the diagonalization of the covariance matrix of the C_α_ atomic fluctuations and depicted in Figure S4 with decreasing order versus the corresponding eigenvector indices. The first few eigenvalues corresponding to concerted motions quickly decrease in amplitude to reach a number of constrained and more localized fluctuation. Exactly, the first two eigenvectors obtained from PC analysis capture more than 61.2% of the total motion, indicating that these vectors define the essential subspace of the system. According to Figure S4, the eigenvalues of two *apo* conformations (Ab1 and Ab2) are higher than the binding conformations (Ab3 and Ab4). This result suggests that properties of motions in four conformations described by the first two PCs are different.

**Figure 1:**
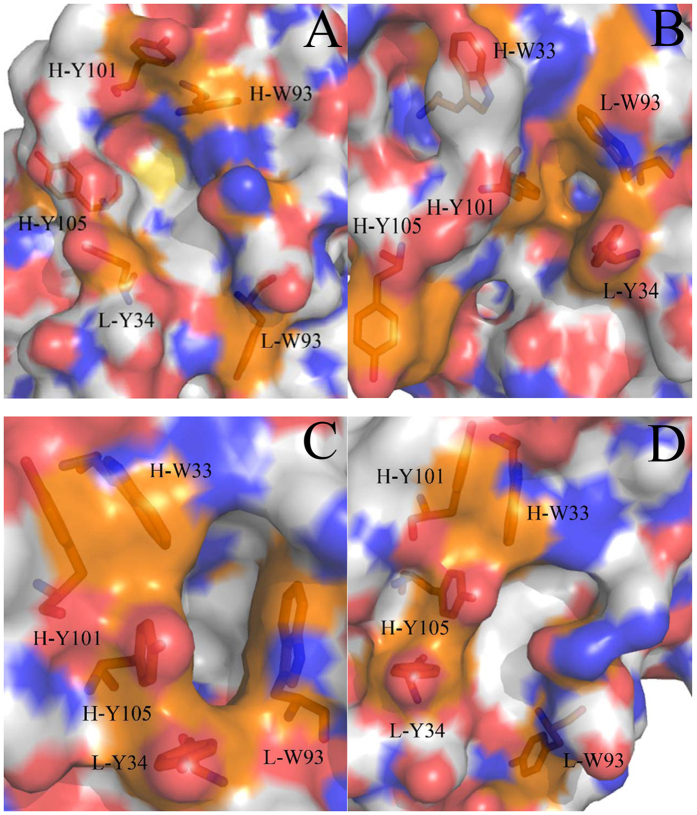
Four different conformations of antibody SPE7 are presented in surface modes. The first capital in labels indicates identification of chains and the second capital represents the name of residues: (**A**) Ab1, (**B**) Ab2, (**C**) Ab3 and (**D**) Ab4.

**Figure 2:**
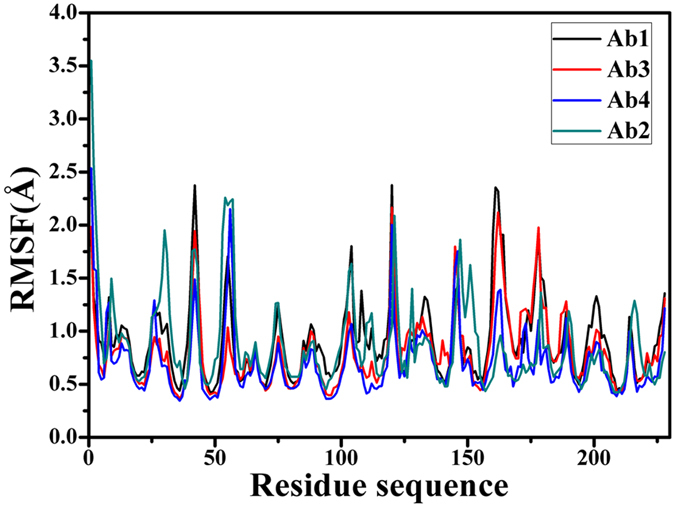
Root-mean-square fluctuations (RMSF) of C_α_ atoms in four conformations Ab1, Ab2, Ab3 and Ab4 of antibody SPE7.

**Figure 3:**
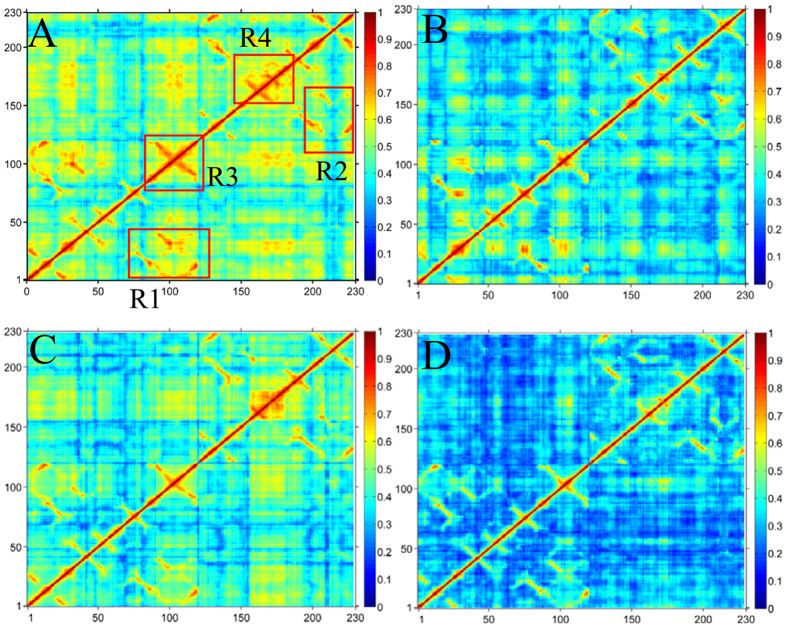
Cross-correlation matrices of fluctuations of the C_α_ atoms around their mean positions from MD trajectories. The extent of correlated motions are represented using color-coded: (**A**) Ab1, (**B**) Ab2, (**C**) Ab3 and (**D**) Ab4.

**Figure 4:**
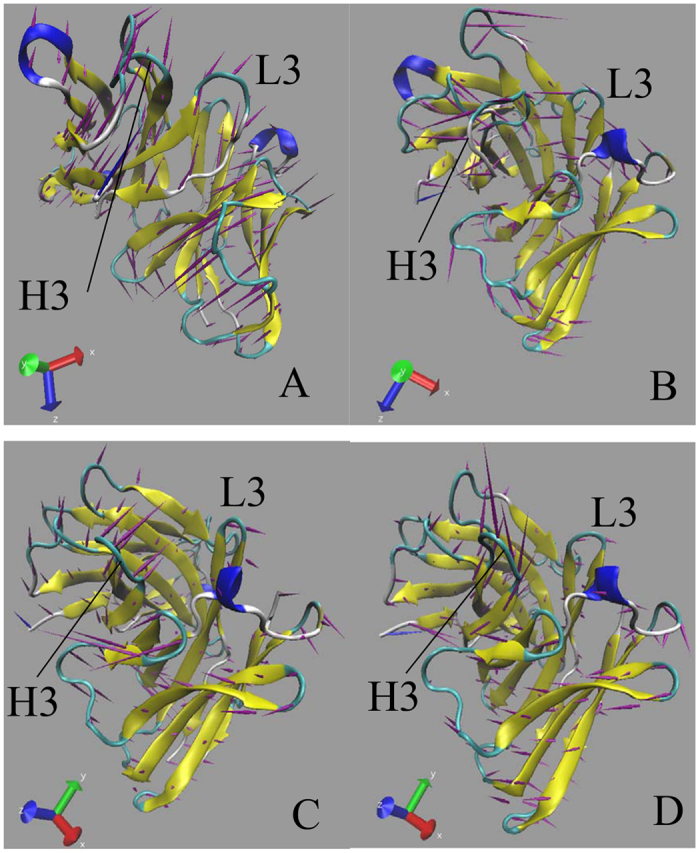
Porcupine plot corresponding to PC1 obtained by performing principal component analysis on MD trajectories: (**A**) Ab1, (**B**) Ab2, (**C**) Ab3 and (**D**) Ab4.

To quantitatively understand the movement directions captured by the eigenvectors, a porcupine plot was generated using the extreme projections on principal component PC1 (Fig. 4). The direction of the arrow in each C_α_ atom represents the direction of motion, while the length of the arrow characterizes the movement strength. The obtained plot suggests that rotational concerted movements are observed in four conformations. The two loops H3 and L3, encircling the binding site of SPE7, displays different motion modes between them. The loops H3 and L3 in the Ab1 move oblique upward in an almost parallel modes (Fig. 4A), and this motion mode may lead to a flatter and shallow channel (Fig. 1A). For the Ab2, the loops H3 and L3 move in an opposite direction and close each other (Fig. 4B), which results in formation of a deep binding site (Fig. 1B). As shown in Fig. 4C, the loop H3 moves oblique upward, but the loop L3 slightly moves oblique downward. This motion mode may encircle a shape of the Ab3 formed in Fig. 1C. For the last conformation Ab4 (Fig. 4D), the loop L3 moves upwards and the loop L3 goes to the right, and their motion directions are almost vertical to each other. This movement mode may induce the conformation shown in Fig. 1D. In addition, Fig. 4 shows that the motion modes of two loops H3 and L3 produce great changes, which agrees well with the changes in cross-correlation analysis.

**Figure 5:**
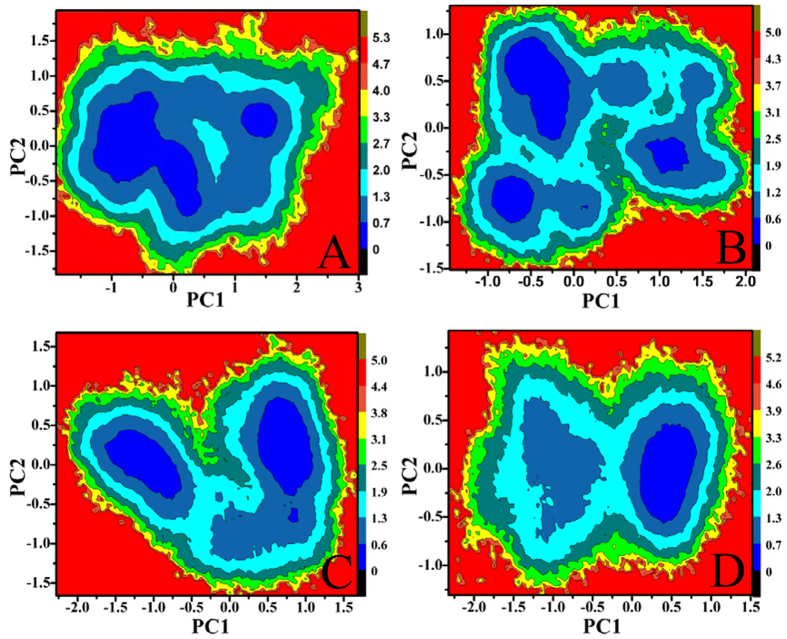
Energy landscape built by using projections of MD trajectories on two eigenvectors corresponding to the first two PCs: (**A**) Ab1, (**B**) Ab2, (**C**) Ab3 and (**D**) Ab4.

**Figure 6:**
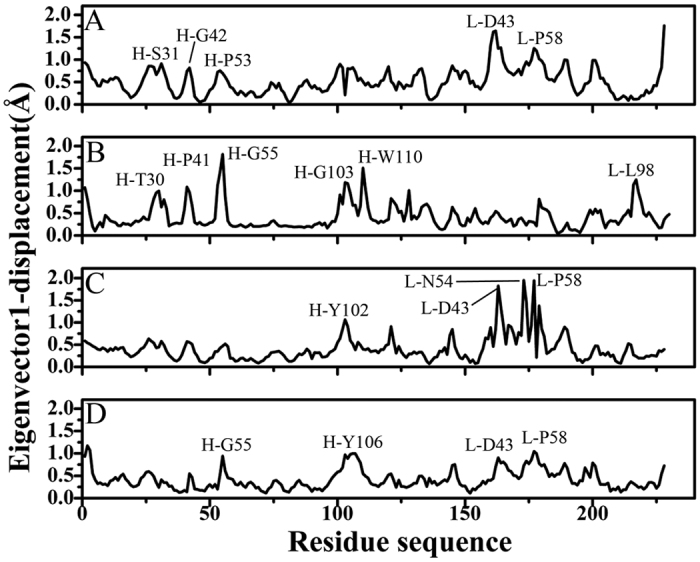
Residue displacements of the first eigenvectors for all four models: (**A**) Ab1, (**B**) Ab2, (**C**) Ab3 and (**D**) Ab4.

Free energy landscapes can provide a valuable information to probe different conformations sampled by the protein in its metastable state. In our studies, free energy landscapes were built by using projections of MD trajectories on two eigenvectors corresponding to the first two PCs (Fig. 5). As seen from Fig. 5, four conformations span different subspace of structures during 200-ns MD simulations. In the Ab1, the protein sequentially visits two conformational regions (Fig. 5A). These regions correspond to enhanced displacements in the H-S31, H-G42, H-P53, L-D43 and L-P58 (Fig. 6A), mainly occurring in some loop regions near the loop H3. The observed large displacements are also indicative for the wide conformational space explored by the residues. In the Ab2, larger displacements occur in the regions near H-T30, H-P41, H-G55, H-G103, H-W110 and L-L98 (Fig. 6B), which in return makes SPE7 frequently visit three conformational regions and span three main subspaces (Fig. 5B). In the presence of AZR, SPE7 mainly visits two conformational regions and crosses two subspaces of structures (Fig. 5C). According to Fig. 6C, the motion of PC1 is observed to correspond to large displacements that are very different from the other three conformations (Ab1, Ab2 and Ab4), especially for the region near L-D43, L-N54 and L-P58. More importantly, these large displacements occur in the chain L except for the residue H-Y102, which may be an important origin inducing the Ab3 conformation of SPE7. For the Ab4, Fig. 5D suggests that both the direction of motion and the probability of visited conformational space differ from the other three conformations. The larger displacements appear in the regions near the residues H-G55, H-Y106, L-D43 and L-P58. Based on the above analysis, the differences in the displacement of residues near the binding site may induce the conformational diversity of SPE7.

### Energy basis of conformational diversity of SPE7

**Figure 7:**
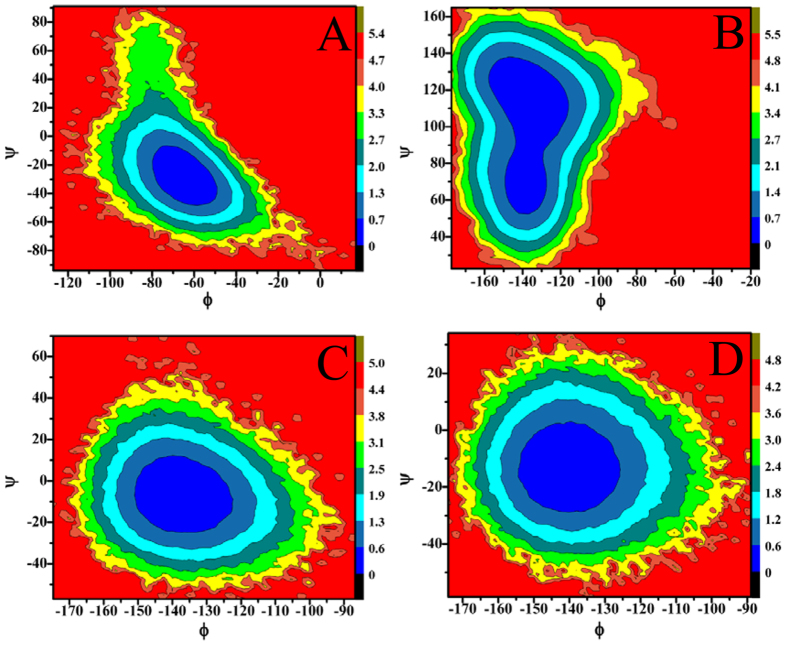
Contour maps of free energies as a function of the backbone angle Φ and Ψ of the residue H-Y101: (**A**) Ab1, (**B**) Ab2, (**C**) Ab3 and (**D**) Ab4.

**Figure 8:**
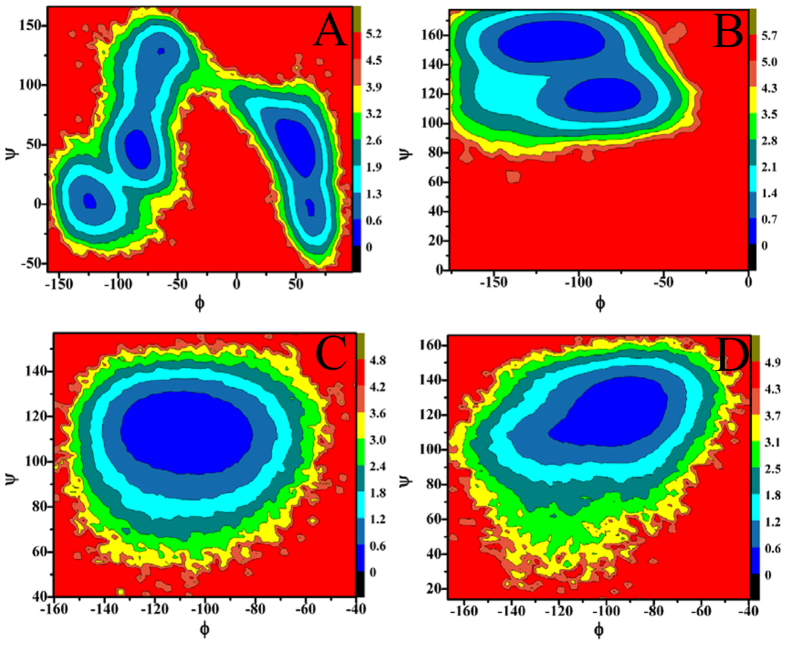
Contour maps of free energies as a function of the backbone angle Φ and Ψ of the residue H-Y105: (**A**) Ab1, (**B**) Ab2, (**C**) Ab3 and (**D**) Ab4.

To reveal energy basis of SPE7 conformational diversity and potential mechanisms inducing cross-reactivity of single antibody toward multiple antigens, free energy landscapes were constructed by using the backbone angles ψ and φ from MD trajectories (Figs 7 and 8). According to Fig. 7, the angles ψ and φ of H-Y101 in the Ab3 and Ab4 do not change obviously, and they mainly correspond to −15° and −135°, respectively (Fig. 7C,D). However, Fig. 7A demonstrates that the angles ψ and φ of H-Y101 are changed into −30° and −60° in the Ab1, respectively, while the ones in the Ab2 are 110° and −140° (Fig. 7B). This result shows that the changes in the angles ψ and φ of H-Y101 may provide special contribution to the conformational diversity of SPE7. Meanwhile, Fig. 8 shows that the angles ψ and φ of the residue H-Y105 display different behavior. Firstly, the angle ψ of H-Y105 is about 110° in the Ab3 and Ab4, but the angle φ exists an obvious difference of about 20° between these two conformations. Secondly, the backbone angle (φ, ψ) in the Ab1 is mainly located at (−75°, 45°) and (25°, 50°), but the backbone angle (φ, ψ) in the Ab2 is distributed at (70°, 150°) and (−75°, 110°). These differences in the angles ψ and φ of H-Y105 may play a certain role in the conformational diversity of SPE7. However, for the residues H-W33, L-Y34 and L-W93, the angles ψ and φ do not produce obvious changes (Figures S5–7), which indicates the angles ψ and φ of these three residues near the binding site do not provide valuable contributions to the conformational diversity of SPE7.

**Figure 9:**
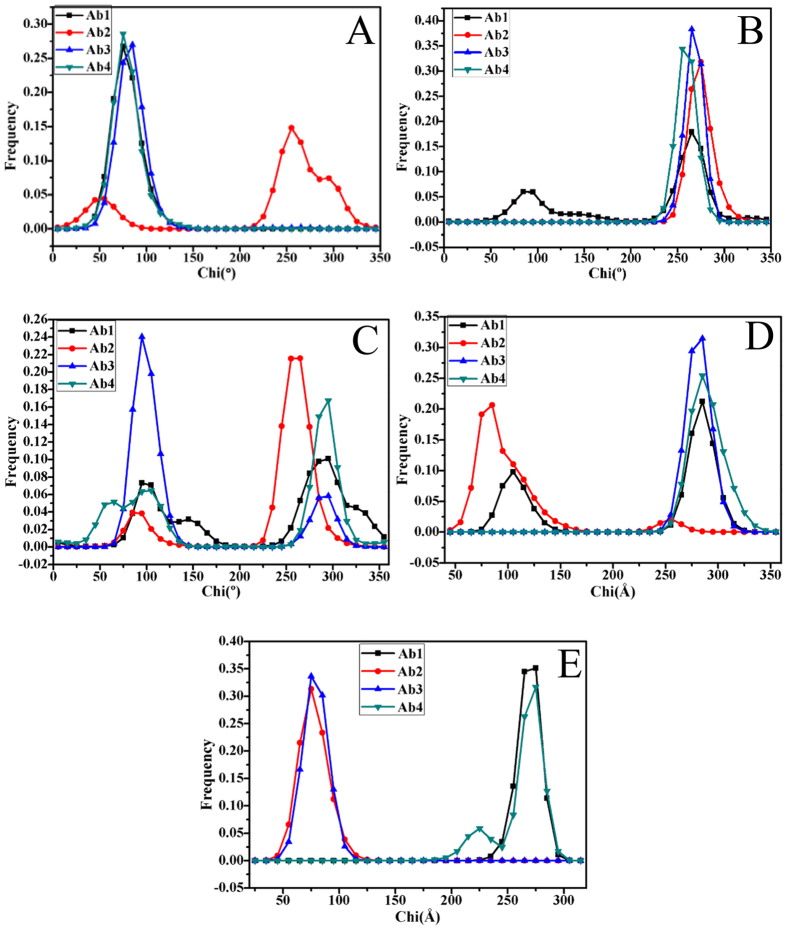
Frequency distribution of dihedral angle from MD trajectories: (**A**) H-W33, (**B**) H-Y101, (**C**) H-Y105, (**D**) L-Y34 and (**E**) L-W93.

**Figure 10:**
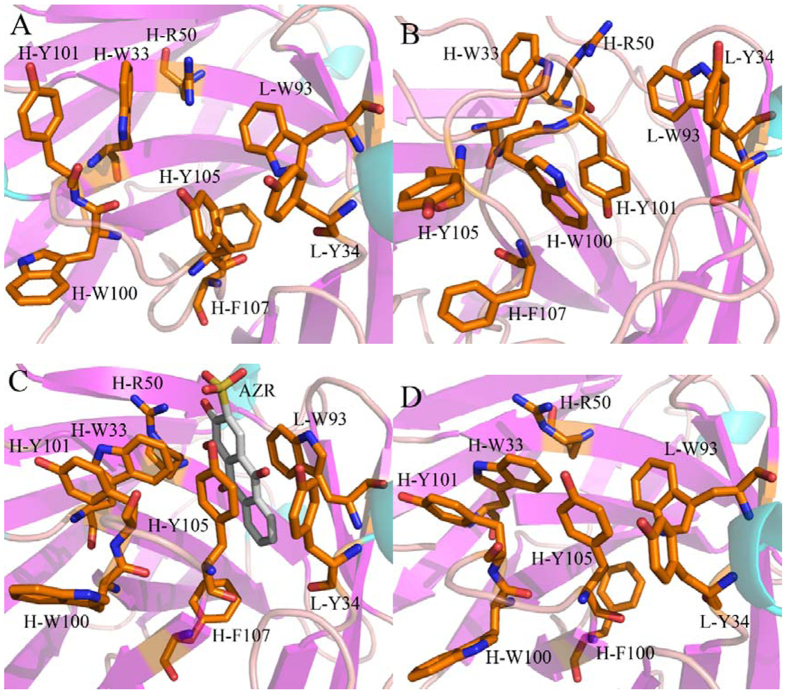
Geometries of key residues near binding fit site of the antibody SPE7 based on the lowest energy level: (**A**) Ab1, (**B**) Ab2, (**C**) Ab3 and (**D**) Ab4.

To evaluate the roles that the conformational changes of residue sidechains play in the conformational diversity of SPE7, the analyses of dihedral angles (chi) were performed on the residues H-W33, H-Y101, H-Y105, L-Y34 and L-W93. Figure 9 gives the frequency distributions of dihedral angles from MD trajectories. Geometries of key residues near the binding site of SPE7 were depicted in Fig. 10. The dihedral angles (chi) of H-W33 in both Ab1 and Ab4 are located near 75°, but this angle in the Ab2 is increased by 180° relative to the Ab1 and Ab4 (Fig. 9A). As a result, the π-π interactions formed between H-W33 and H-Y101 or H-Y105 are lost in the Ab2 (Fig. 10B). The chi angle of H-W33 in the Ab3 is only rotated an angle of 10° relative to the Ab1 and Ab4 (Fig. 9A), the π-π interactions between H-W33 and H-Y101 or H-Y105 are maintained (Fig. 10A,C,D). According to Fig. 10B, the chi angle of H-Y101 in four conformations does not generates obvious change, which is supported by the relative orientation of H-Y101 in four conformation (Fig. 10). Thus, the side chain’s orientation of H-Y101 does not provide important contribution to the conformational diversity of SPE7. As shown in Fig. 9C, the chi angles of H-Y105 in the Ab1 and Ab4 are mainly located at ~295° and the π-π interactions of H-Y105 with H-W33 and L-Y34 can be maintained in this orientation, which possibly leads to a shallow conformation in the Ab1 and Ab4 (Fig. 10A,D). However, the dihedral angle of H-Y105 in the Ab2 is rotated to 258° and its side chain goes toward the deep of protein, which will lead to the deeper channel of the Ab2 (Fig. 10B). Figure 9C shows that the chi angle of H-Y105 in the Ab3 is changed into 95°. This orientation make its side chain form a vertical angle with the residue H-W33 to produce strong π-π interactions with AZR, which favors the formation of a deep foot-shape conformation in the Ab3 (Fig. 10C). Thus, it is reasonably concluded that the change in the side chain of H-Y105 provides a key contribution to the conformational diversity of SPE7. According to Fig. 9D, the dihedral angle of L-Y34 in the Ab1, Ab3 and Ab4 are mainly distributed at 285°, while the one in Ab2 is near 84°. As a result, the residue L-Y34 involves an edge part of the binding site in the conformation Ab1, Ab3 and Ab4 (Fig. 10A,C,D), but leads to a deep channel encircled by H-Y105 and L-Y34 in the Ab2 (Fig. 10B). Figure 10E suggests that the chi angle of the residue L-W93 in the Ab1 and Ab4 is ~276°, while the one in the Ab2 and Ab3 is ~75°. This positions are easy to produce the shallow channel in the Ab1 and Ab4, also help to form a deeper binding site in the Ab2 and Ab3. This result indicates that the alternation of side chain in L-W93 may play an important role in the conformational diversity of SPE7.

Based on the above analyses, the backbone angles ψ and φ in the residues H-Y101 and H-Y105 are obviously different from the other residues, which provides partial contributions to the conformational diversity of antibody SPE7. The dihedral analyses of sidechain in key residues encircling the binding site of SPE7 show that the changes in the chi angle of four residues H-W33, H-Y105, L-Y34 and L-W93 produce significant effect on the structure of SPE7. Compared to the backbone angle ψ and φ, the alternation of dihedral angles of key residues provide more important contribution to the conformational diversity of SPE7. It is concluded that rearrangement of side chains of residues in several key positions induce the conformational diversity of SPE7. These different conformations may generate an ability to bind with different antigens, which gives a reasonable explanation to the cross-reactivity of SPE7 or the relationship of single antibody toward multiple antigens.

## Discussion

In this work, a serials of 200-ns MD simulations were carried out to probe potential molecular mechanisms and energy basis of single antibody toward multiple antigens and clarify the reasons inducing cross-reactivity of antibody SPE7. Compared to standard MD simulations, umbrella sampling or metadynamics simulations can enhance conformation sampling and ensure reliability of the simulated results. However, because of big difference in structures of two antigens, it is not easy to determine the appropriate reaction coordinates of four simulated systems. Thus, standard MD simulations were used as the studying tools of current work. To ensure the convergence of MD trajectories, we performed two separate MD simulations on four conformations with different random seeds and some corresponding analyses were listed in Figures S8 and S9. The obtained similar results show that our MD simulations are convergent and the current obtained results are reliable. The free energy landscapes between RMSD and gyration radius demonstrate that four conformations Ab1, Ab2, Ab3 and Ab4 have different energy levels, which implies that four conformations have different internal dynamic behavior during MD simulations.

The followed PC analysis was performed to reveal the origin of large conformational difference exists between the loops H3 and L3, which is supported by their different motion direction relative to each other. Analyses of residue displacements based on projection of MD trajectories on the first component proved that larger displacements of some key residue make four conformations visit different subspace during MD simulations, which gives significant dynamics information involving conformational diversity of antibody SPE7.

The free energy landscapes were constructed by using the backbone angles ψ and φ from MD trajectories to obtain energy information of SPE7 conformational diversity. The results suggest that the angle changes of the residues H-Y101 and H-Y105 play a certain role in the conformational diversity of SPE7. However, more significant origin of the conformational diversity of SPE7 should stem from the side-chain conformational changes of several key residues H-W33, H-Y105, L-Y34 and L-W93, which has been proved by the dihedral angle analyses of the residues near the binding site based on MD simulations.

The dihedral angle analyses of residues around the binding site also reveal that the side-chain rearrangement of key residues significantly affect the inter-residue hydrophobic interactions such as the π-π interactions. These interaction changes give a hint that more attentions should be paid to the hydrophobic interactions of inhibitors with SPE7 as well as how to optimize these interactions during the development of potent drugs curing the disease related with SPE7. We also expect that this study can provide a theoretical guidance for understanding the mechanism of cross-reactivity for SPE7 and antibody-drug conjugated anti-disease treatment.

## Methods

### System preparation

The crystal structures of four different conformations of antibody SPE7 were obtained from the protein data bank (PDB): 1OAQ for the *apo* Ab1 conformation, 1OAW for the *apo* Ab2 conformation, 1OAR for the Ab3 conformation complexed AZR and 1OAZ for the Ab4 conformation complexed a recombinant protein antigen (Trx-Shear3)^3^. The protonated states of residues were checked by using the program PROPKA^56^. All crystal water molecules were kept in simulation system. The general Amber force field (GAFF)^57^ and AM1-BCC charges were assigned to AZR by using the Leap module and the antechamber module. The amber ff99SB force field was adopted to generate the force field parameters of proteins and water molecules^58^. Each protein or complex was solvated in a cubic box of TIP3P water molecules^59^, keeping the boundary of the box at least 14.0 Å away from any solute atom. Counterions were added to neutralize each system.

### MD simulations

In this work, all simulations were performed by using the program GROMACS^60^ version 4.5.3 based on the NPT ensemble and periodic boundary condition. Firstly, to remove some bad contacts formed by the preparation of system, each system was subjected to energy minimization using the steepest descent algorithm. Secondly, a 5 ns MD simulation was run to heat the system from 0 K to 300 K by fixing the solute with a harmonic restraint of force constant of 10 kcal·mol^−1^ Å^−2^, followed by another 5 ns MD simulation with the protein C_α_ atoms and small molecule restrained. Finally, the 200-ns production phase was carried out without any constraints to relax each system. The bond lengths involving hydrogen atoms was restrained by using the LINCS method^61^ so that a longer integration step of 2 fs can be adopted. A distance cutoff of 12.0 Å was applied to compute non-bond interactions involving van der Waals and electrostatic interactions. The electrostatics interactions were calculated by using the particle Mesh Ewald (PME) algorithm^62^. The temperature of the system was kept at 300 K using a novel V-rescale thermostat with a response time of 1.0 ps^63^. The pressure was maintained at 1 bar using the Parrinello-Rahman pressure coupling scheme^64^.

### Principal component analysis

In current work, the internal collective motions of antibody SPE7 were also explored by using the positional covariance matrix *C* constructed using the coordinates of C_α_ atoms and its eigenvectors of *C*^65^,^66^. The elements of the positional covariance matrix *C* were obtained based on the equation 1.


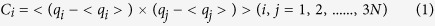


The quantity *q*_*i*_ is the Cartesian coordinate of the *i*th C_α_ atom, and *N* is the number of C_α_ atom used in building matrix *C*. The averaged values are computed over the equilibrated phase of MD simulations after superimposition on a reference structure to remove overall translations and rotations by using a least-square fit procedure^47^,^67^. The matrix *C* is symmetric and can be diagonalized by an orthogonal coordinate transformation matrix *T* which can transform the matrix *C* into a diagonal matrix *Q* of eigenvalues *λ*_*i*_:


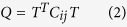


in which each column represents the eigenvector that describes the direction of motion relative to <*q*_*i*_>, and each eigenvalue exhibits the total mean-square fluctuation of the system along the corresponding eigenvector. To probe internal dynamics of conformational diversity for SPE7, cross-correlations between C_α_ atoms of protein in four simulation systems were calculated using the generalized correlation analysis approach developed by Lange and Grubmüller^53^. The g_correlation module in the GROMACS package was used for the analysis.

### Energy landscapes

An energy landscape is a mapping of all possible conformations of a molecular entity, and it plays an important role in probing the spatial position of interacting molecules in a system and gives their corresponding energy levels^52^,^68^. To reveal energy bases of SPE7 conformational diversity, energy landscapes between different quantities were calculated according to the following equation.


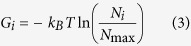


where *k*_*B*_ is Boltzmann’s constant, *T* is the temperature of simulation systems and 300 K is set in the current calculations. *N*_*i*_ is the population of bin *i* and *N*_max_ is the population of the most populated bin. Bins with no population are given an artificial barrier scaled as the lowest probability. Different energy levels are displayed using color-code modes.

## Additional Information

**How to cite this article**: Chen, J. *et al*. Molecular Mechanism and Energy Basis of Conformational Diversity of Antibody SPE7 Revealed by Molecular Dynamics Simulation and Principal Component Analysis. *Sci. Rep.* **6**, 36900; doi: 10.1038/srep36900 (2016).

**Publisher's note:** Springer Nature remains neutral with regard to jurisdictional claims in published maps and institutional affiliations.

## Supplementary Material

Supplementary Information
