# Supplementary material for: Molecular Mechanism and Energy Basis of Conformational Diversity of Antibody SPE7 Revealed by Molecular Dynamics Simulation and Principal Component Analysis
Source: Sci Rep. 2016 Nov 10;6:36900. doi: 10.1038/srep36900 (PMC5103278; doi:10.1038/srep36900)
Supplement: Supplementary Information [file srep36900-s1.pdf]

Molecular Mechanism and Energy Basis of Conformational Diversity of Antibody SPE7 Revealed by Molecular Dynamics Simulation and Principal Component Analysis

Jianzhong Chen[a], Jinan Wang[b] and Weiliang Zhu[b]

[a]School of Science, Shandong Jiaotong University, Jinan, 250014, China.

[b]Drug Discovery and Design Center, CAS Key Laboratory of Receptor Research, Shanghai Institute of Materia Medica, Chinese Academy of Sciences, 555 Zuchongzhi Road, Shanghai, 201203, China

Corresponding Author

*E-mail: J.C. (chenjianzhong1970@163.com), J.W. (wangjinanzhl@sina.com),

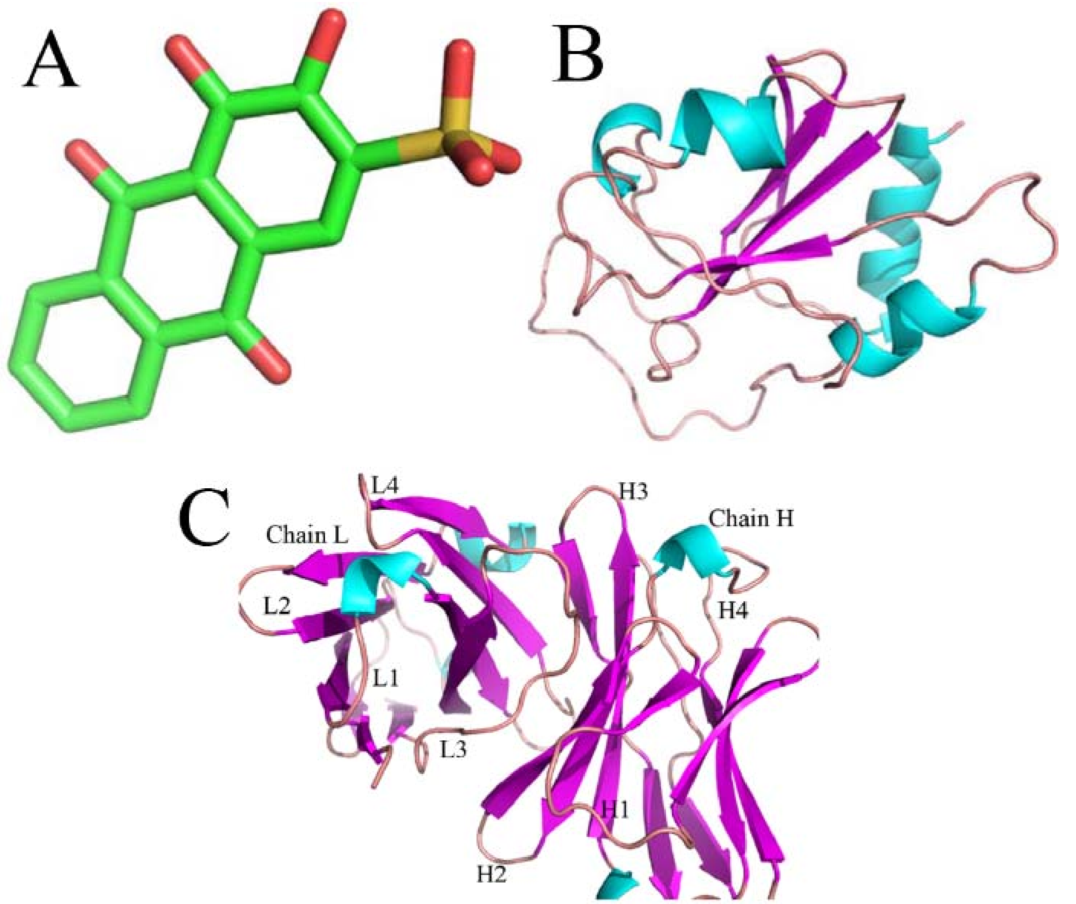

Figure S1 structures of AZR, antigens Trx-Shear3 and antibody SPE7: (A) AZR, (B) Trx-Shear3 and (C) antibody SPE7.

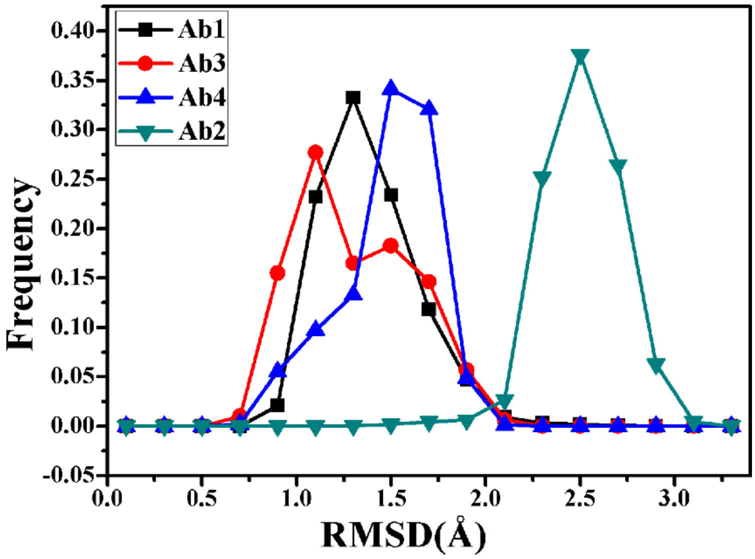

Figure S2 Frequency distribution of root-mean-square deviations (RMSD) of the backbone atoms relative to the corresponding crystal structures

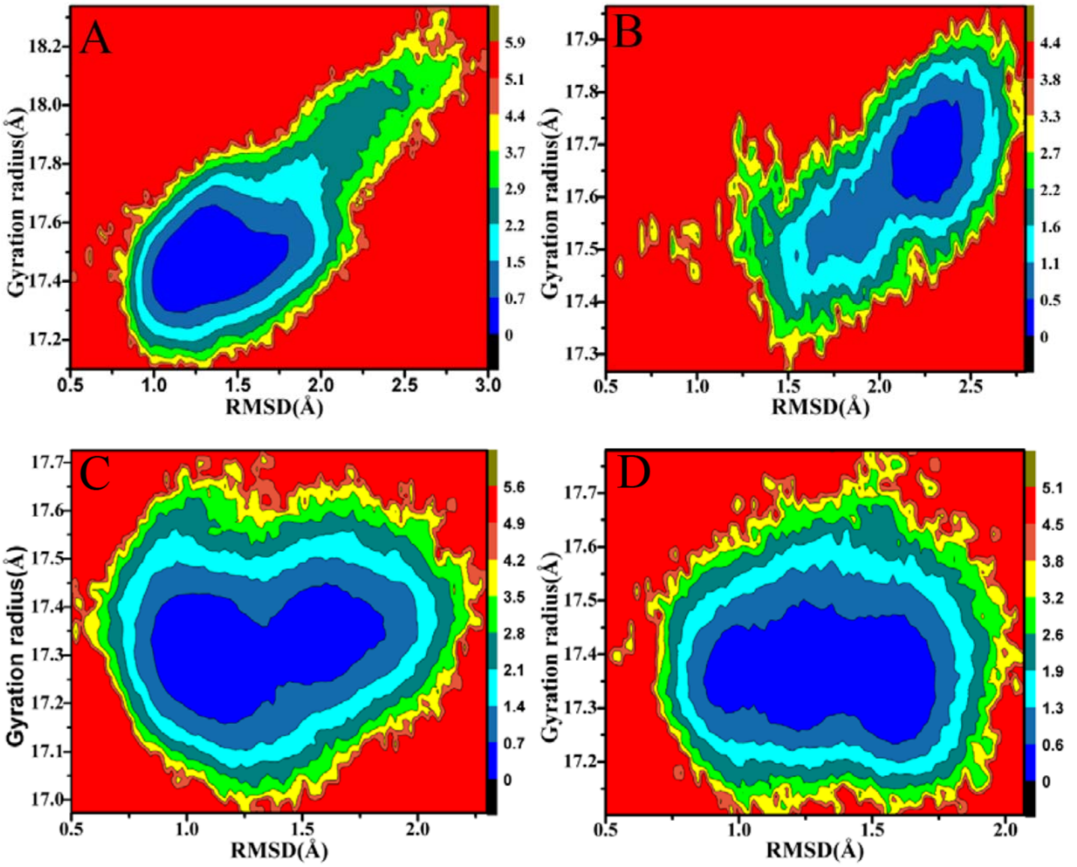

Figure S3 Free energy landscape of four different conformations Ab1, Ab2, Ab3 and Ab4 built by using RMSD and gyration radius: (A) Ab1, (B) Ab2, (C) Ab3 and (D) Ab4.

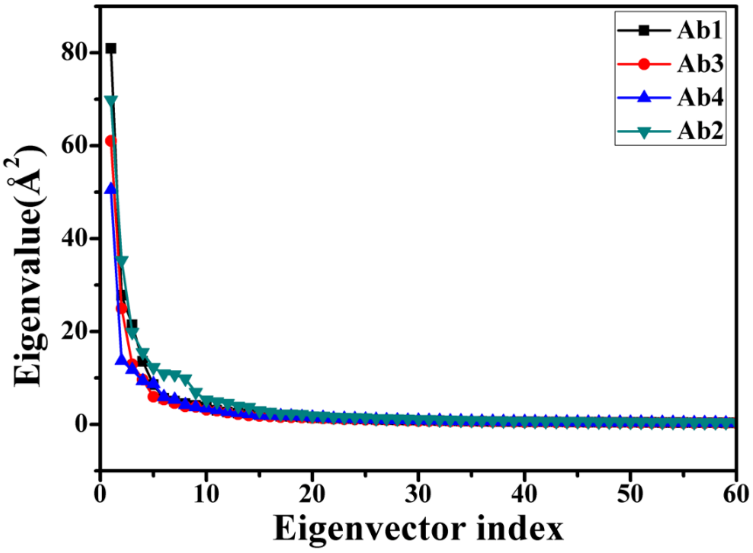

Figure S4 Comparison of the eigenvalues plotted against the corresponding eigenvectors indices obtained from the Cα covariance matrix built from MD trajectories.

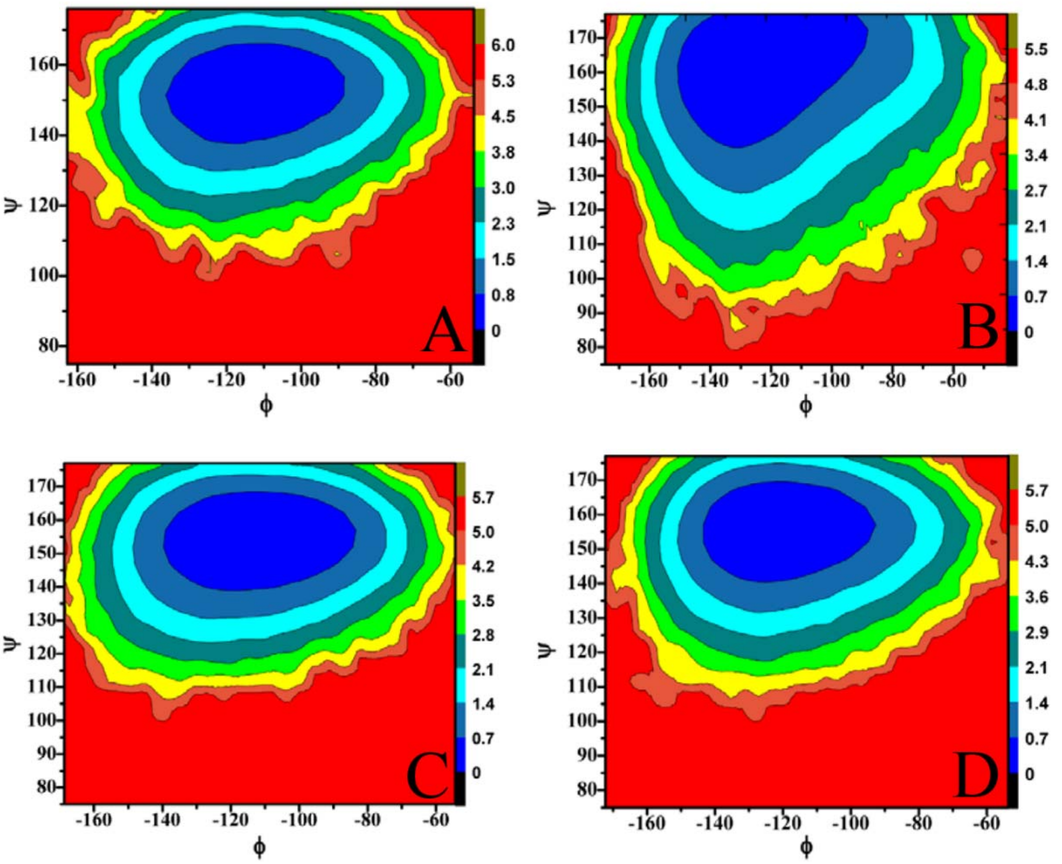

Figure S5 Energy landscapes constructed by using the backbone angle of the residue H-W33: (A) Ab1, (B) Ab2, (C) Ab3 and (D) Ab4.

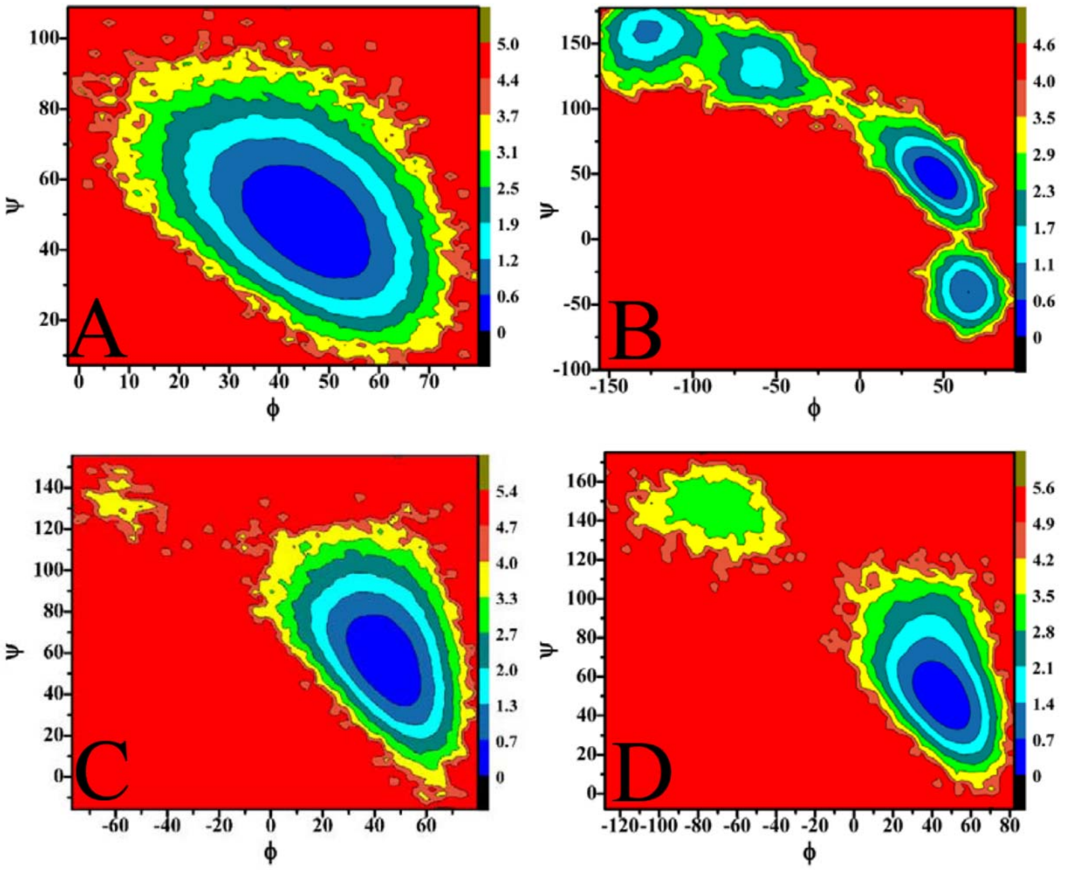

Figure S6 Energy landscapes constructed by using the backbone angle of the residue L-Y34: (A) Ab1, (B) Ab2, (C) Ab3 and (D) Ab4.

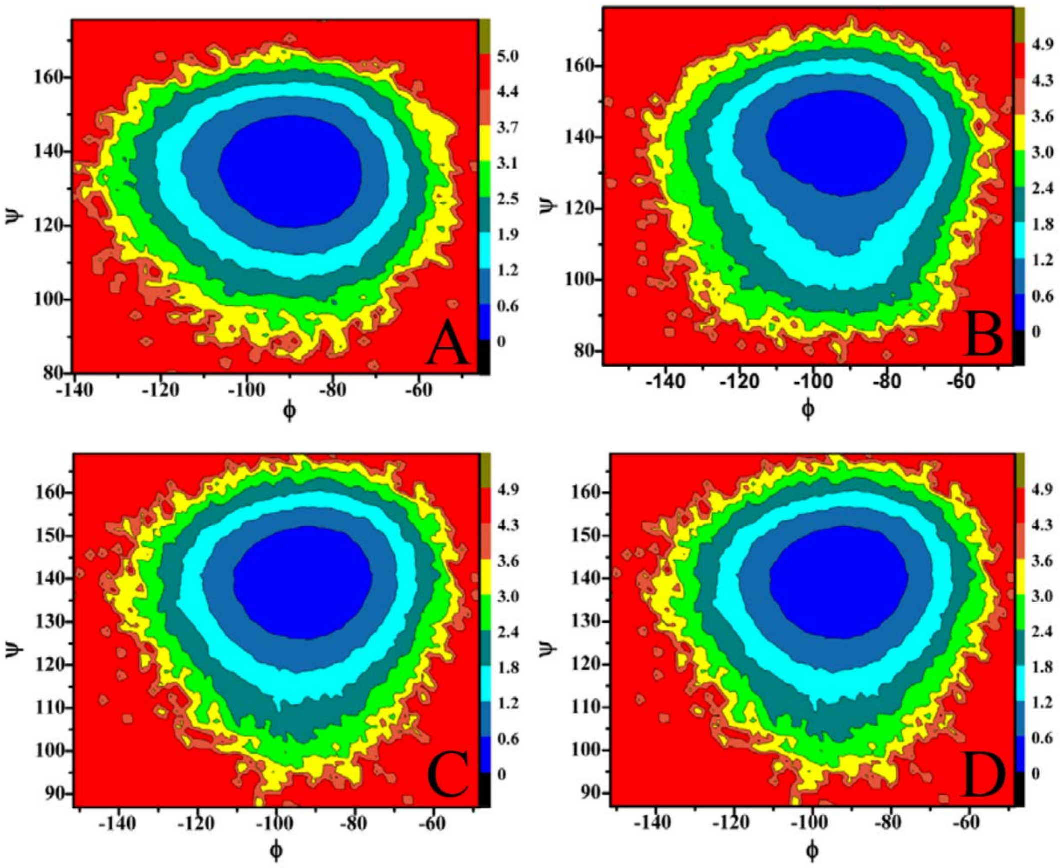

Figure S7 Energy landscapes constructed by using the backbone angle of the residue H-W33: (A) Ab1, (B) Ab2, (C) Ab3 and (D) Ab4.

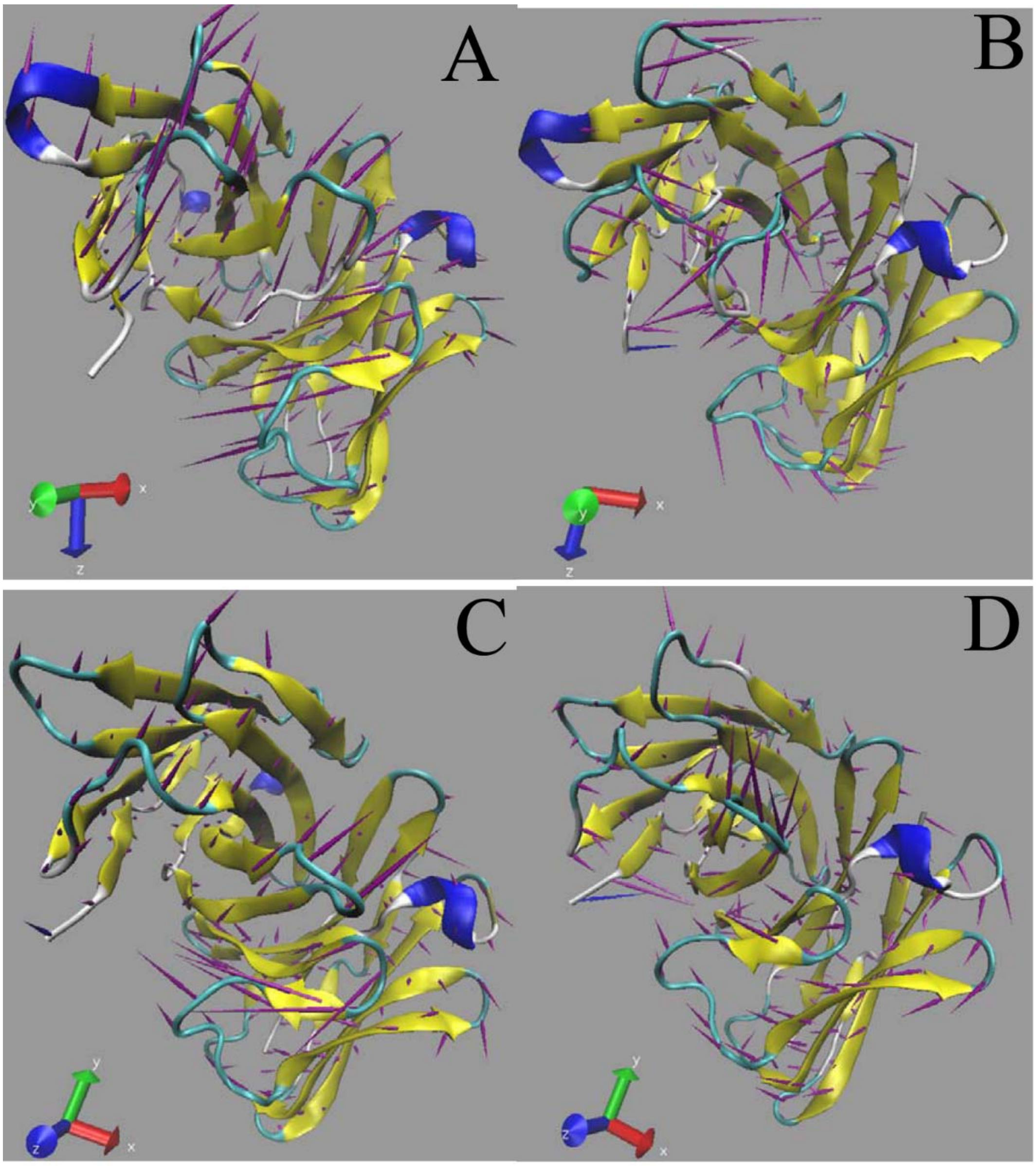

Figure S8 Porcupine plot corresponding to PC1 obtained by performing principal component analysis on new MD trajectories: (A) Ab1, (B) Ab2, (C) Ab3 and (D) Ab4.

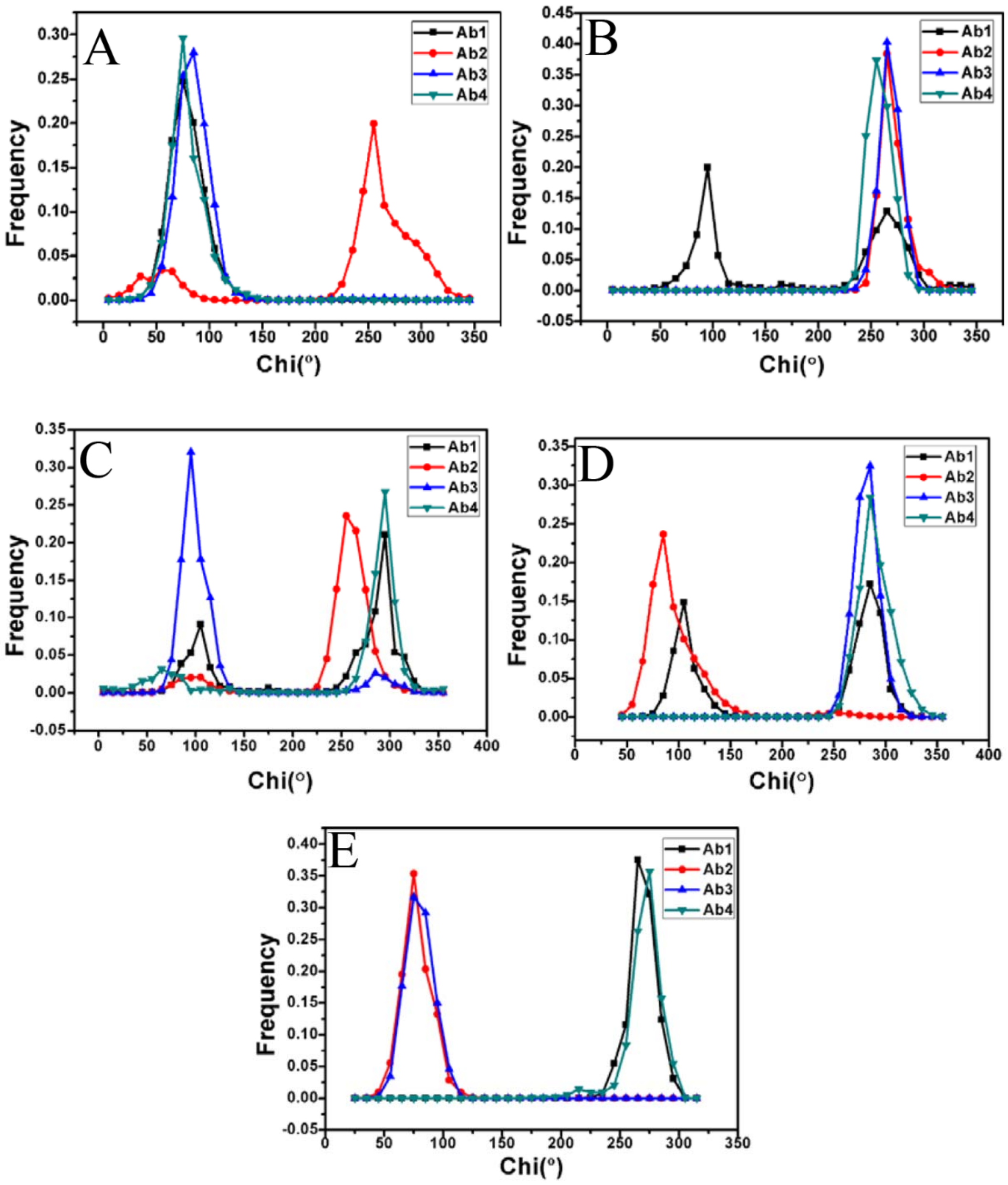

Figure S9 Frequency distribution of dihedral angle by using new MD trajectories: (A) H-W33, (B) H-Y101, (C) H-Y105, (D) L-Y34 and (E) L-W93.
